# Effect of Intravenous Patient Controlled Ketamine Analgesiaon Postoperative Pain in Opium Abusers

**DOI:** 10.5812/aapm.14129

**Published:** 2014-02-15

**Authors:** Mastane Dahi-Taleghani, Benjamin Fazli, Mahshid Ghasemi, Maryam Vosoughian, Ali Dabbagh

**Affiliations:** 1 Anesthesiology Research Center, Shahid Beheshti University of Medical Sciences, Tehran, Iran

**Keywords:** Ketamine, Acute Pain, Opium, Drug Users

## Abstract

**Background:**

Acutepostoperative pain is among the worst experience that patient scan undergo, and many analgesics have been used to suppress it; especially in chronic opium abusers. Ketamine is an N-methyl-D-aspartate antagonist analgesic, having both anesthetic and analgesic properties, which are not affected to the same extent in chronic opium abusers.

**Objectives:**

In this study, we assessed the analgesic effects of ketamine added to morphine as a patient-controlled analgesia method for acute pain management, compared with a placebo, inchronic maleopium abusers.

**Patients and Methods:**

After institutional review board approval for ethical considerations, a randomized double-blinded placebo controlled clinical trial was conducted. A total of 140 male patients aged 18-65 years, undergoing orthopedic surgery, were entered into the study after matching inclusion and exclusion criteria. All patients received the same anesthesia method; while the first group received ketamine (1mg/mL) and morphine (0.5 mg/mL) as a patient-controlled analgesia (70 patients), the second group received morphine (0.5 mg/mL) plus normal saline (70 patients). P value less than 0.05 was considered statistically significant.

**Results:**

The ketamine and morphine group of patients experienced less postoperative pain and required less postoperative rescue analgesia. However, the unwanted postoperative side effects were nearly the same; although increased levels of postoperative nausea and vomiting were observed in the ketamine and morphine group

**Conclusions:**

This study demonstrated improved analgesic effects after using intravenous patient controlled analgesia with ketamine on postoperative pain in opium abusers.

## 1. Background

Acute postoperative pain is one of the most difficult experiences that patients must tolerate. Many different analgesics have been used; each having their own special drawbacks (1). Among patients with acute pain, chronic opium abusers are a real challenge (2-5), as they usually need extremely high doses of analgesics for acute pain suppression, and in addition, some analgesics are not very effective on them (6, 7). To resolve this problem, many analgesics have been tested with controversial results.

Ketamine is an N-methyl-D-aspartate antagonist (8-10), and it is mainly used as an anesthetic with simultaneous analgesic properties. Since it is an NMDA antagonist and its analgesic properties do not stimulate opioid receptors, it induces analgesia through NMDA receptors instead, which is a more effective mechanism in opium abusers (10-18). Although there are some controversies regarding ketamine (19-22), recent clinical studies have demonstrated its effectiveness as an analgesic agent in opium abusers (11). However, the analgesic effects of ketamine in improving clinical outcomes in opium abusers, especially when used as a patient controlled analgesia (PCA), are not well defined and need more research (23).

## 2. Objectives

In this study, we assessed the analgesic effects of ketamine when added to a morphine sulfate solution as a PCA method for acute pain management, compared with a placebo in male patients with a history of chronic opium abuse.

## 3. Patients and Methods

This was a randomized double-blinded placebo controlled clinical trial. The proposal of the study was approved for ethical considerations by the institutional review board of Shahid Beheshti University of Medical Sciences, Tehran, Iran. Furthermore, the manuscript was approved by the Ethics Committee, Research Deputy, Shahid Beheshti University of Medical Sciences and the following comments were followed throughout all stages of the study:

1) The study protocol conforms to the ethical guidelines of the 1975 Declaration of Helsinki.

2) Informed written consent was taken from each patient separately, before entering the study

### 3.1. Patient Selection and Sample Size Calculation

**Figure 1.**
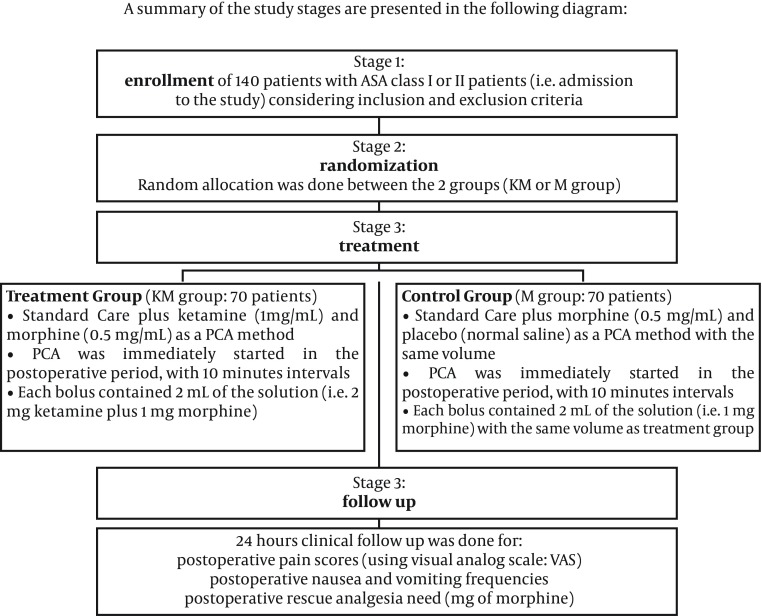
Summary of the Study Stages are Presented in the Diagram

All male patients, aged 18-65 years undergoing orthopedic surgery in a university hospital, were considered as the target population. After considering the inclusion and exclusion criteria and calculating the sample size, the patients entering the study were selected. The inclusion criteria consisted of; male gender, elective orthopedic surgery of the lower limb, history of opium abuse (inhalational or oral routes, on a regular basis for at least two years, leading to drug craving behaviors in case of drug deprivation, all based on the patient's declaration), application of general anesthesia, no contraindication for general anesthesia, and having no significant co-morbid disease (i.e. all were American Society of Anesthesiology (ASA) class I or II patients). The exclusion criteria included; female gender, other anesthetic methods (except for general anesthesia), ASA class more than III, duration of anesthesia less than one hour, other routes of drug abuse (except for inhalational opium for two years), and patient's refusal to continue the study after primary approval for study entry. The patients were then randomly assigned to one of the two study groups based on a computer table of random numbers:

1) The first group consisted of 70 patients who received ketamine plus morphine (KM group) as a PCA method. In this group, a combined solution of 1 mg/mL ketamine and 0.5 mg/mL morphine was prepared as the PCA analgesia protocol. This was started immediately in the postoperative period, at 10 minutes intervals, and each bolus contained 2 mL of the solution (i.e. 2 mg ketamine plus 1 mg morphine).

2) The second group also consisted of 70 patients, and they received a combination of morphine (0.5 mg/mL) plus normal saline solution (M group). Again, PCA analgesia was started immediately in the postoperative period at 10 minutes intervals, using 2 mL of the solution in each PCA bolus (i.e. 1 mg morphine in each bolus). A brief presentation of these stages is demonstrated in Figure 1. 

Sample size determination was done after a power analysis (power = 0.8; β = 0.2; α = 0.02) using sample size software (PASS 2005, NCSS LLC, Kaysville, Utah, USA). The observed frequency for acute postoperative pain in similar patients requesting analgesics was also considered as the clinical criterion for determination of the sample size, and based on the following data and sample size equation (24-27):

n = 2[(Z_β_+Z_α_) σ /Δ]^2^

Where:

- n (sample size in each group) = 62
- α = 0.05
- Zα = 1.96
- β = 0.2
- Zβ = 0.84
- σ (estimated standard deviation based on similar studies) = 2
- Δ = the estimated effect size (i.e. the minimal difference desired between the two study interventions or the clinical outcomes of the two groups)

Considering a possible 10% dropout, 70 patients were considered as the sample size for each study arm, and consequently total number of 140 patients were selected and entered the study after matching the inclusion and exclusion criteria as follows.

### 3.2. Clinical Management of Patients

All patients were visited the night before surgery and the study protocol was described to them. This visit was the task of the authors ‘colleague. He explained the process of the study and took informed written consents for entering the study during this visit. He also explained that entering the study was a completely voluntary issue and any patient entering the study could exit the research process at any time during the study period. In addition, acceptance or refusal to enter the study, or refusal to continue, had no effect on the treatment process (including the treatment of acute pain).

The patients underwent general anesthesia for elective lower limb orthopedic surgery. After entering the operating theatre their vital signs were monitored using; electrocardiogram, pulse oximeter, end tidal CO_2_, and noninvasive blood pressure. For each patient, two intravenous lines were inserted for the administration of fluids, blood and drugs, all according to the study's protocol and the patient's needs. Anesthesia was induced using 0.2 mg/Kg IV midazolam, 200 μg fentanyl, 5 mg/Kg sodium thiopental, and 5 mg/Kg atracurium. Before anesthesia induction, 10 ml/Kg of Ringer's solution was administered freely to compensate for NPO time deficit. For maintenance of the anesthesia, 0.6-1% isoflurane was used to keep the anesthesia depth between 40-60, using Bispectral Index (BIS) monitoring. During final skin suturing, isoflurane was discontinued, and the patients were reversed pharmacologically, using 0.05 mg/kg neostigmine plus 0.02 mg/Kg atropine. After full muscle force recovery and an appropriate level of consciousness, the patients were extubated. Ketamine was not used for anesthesia induction or maintenance at any time during the study. The patients were transferred to the postanaesthetic care unit (PACU) for hemodynamic control, and regaining a full level of consciousness before starting acute pain control remedies.

During the postoperative period, the patients' postoperative pain scores were controlled using a visual analog scale (VAS), and their respiratory status; using pulse oximetry and respiratory rates, were measured. Postoperative pain was documented and registered at 1, 6 and 24 hours after the operation. In addition the patients received supplementary nasal oxygen, if they had any decline of oxygen saturation (SpO_2_) below 92%. However, postoperative pain scores greater than 3 out of 10, mandated postoperative pain control using analgesics through the following protocol:

1. Starting PCA as defined in the patient groupings (KM group vs. M group).
2. Respiratory monitoring including pulse oximetry.
3. Adding supplementary nasal oxygen if SpO_2_ was less than 92%.
4. Administration of supplementary intravenous morphine to each of the patients having a VAS score more than 3 out of 10 in the postoperative period, regardless of their study group.
5. Calculating the extra morphine doses in each patient during the first 24 hour period after the operation.
6. Assessment of postoperative nausea and vomiting frequencies in each patient in the first 24 hour period after the operation.

Data were collected and analyzed using SPSS software, (version 16). A Student's t-test was used for data analysis. P value less than 0.05 were considered statistically significant.

## 4. Results

The two study groups had no significant difference regarding baseline demographic data (Table 1). While the results of postoperative pain scores were lower in the KM group, they also experienced less postoperative pain, and needed less postoperative rescue analgesia (Tables 2 and 3) These results suggest a greater analgesic effect of the PCA solution used in the KM patient group. However, the unwanted postoperative side effects were very similar; although increased postoperative nausea and vomiting was observed in the KM group (Table 4). 

**Table 1.** Baseline Demographic Data in the Two Study Groups

	KM Group ^a^, Mean ± SD, (n = 70)	M Group ^a^, Mean ± SD, (n = 70)	P value
**Age, y**	39.1 ± 7.2	38.3 ± 7.5	> 0.05
**Weight, kg**	68.8 ± 12.5	69.6 ± 11.1	> 0.05
**Operation time, min**	98 ± 18	94 ± 20	> 0.05
**Anesthesia time, min**	132 ± 24	126 ± 26	> 0.05

^a^ KM group; 1mg/mL ketamine and 0.5 mg/mL morphine solution; M group; 0.5mg/mL morphine plus normal saline solution.

**Table 2.** Postoperative Pain Scores in the Two Study Groups

	KM Group ^a^, Mean ± SD, (n = 70)	M Group ^a^, Mean ± SD, (n = 70)	P value
**VAS Score 1 hour After Surgery**	2.4 ± 1.1	3.6 ± 1.3	0.01
**VAS Score 6 hours After Surgery**	1.5 ± 0.8	2.2 ± 1.1	0.03
**VAS Score 24 hours After Surgery**	1 ± 0.5	1.7 ± 0.8	0.02

^a^ KM group; 1mg/mL ketamine and 0.5 mg/mL morphine solution; M group; 0.5mg/mL morphine plus normal saline solution.

**Table 3.** Postoperative Rescue Analgesia in the Two Study Groups

	KM Group ^a^, Mean ± SD, (n = 70)	M Group ^a^, Mean ± SD, (n = 70)	P value
**Morphine, mg (Total 24 Hourdose)**	12 ± 3	7 ± 2	0.01

^a^ KM group; 1mg/mL ketamine and 0.5 mg/mL morphine solution; M group; 0.5mg/mL morphine plus normal saline solution.

**Table 4.** Postoperative Unwanted Side Effects in the Two Study Groups

	KM Group ^a^, Frequency (%), (n = 70)	M Group ^a^, Frequency (%), (n = 70)	P value for Chi Square
**Nausea**	10 (14)	4 (6)	0.03
**Vomiting**	6 (8.5)	1 (1.5)	0.02
**Patients requiring an antiemetic**	8 (11.5)	3 (4)	0.02

^a^ KM group; 1mg/mL ketamine and 0.5 mg/mL morphine solution; M group; 0.5mg/mL morphine plus normal saline solution.

## 5. Discussion

The results of this study demonstrated improved analgesic effects of intravenous patient controlled analgesia with ketamine on postoperative pain in opium abusers. The effects of chronic opium abuse in altering the threshold of pain tolerance have previously been studied in a number of studies since the issue became a high priority in acute pain research (2, 4, 6, 7, 28-32). On the other hand, among this large list, there are not many studies that have assessed the effects of ketamine in postoperative pain suppression in these patients (17, 33). 

There is no doubt that acute pain treatment in such patients is a real challenge (33, 34) and the usual pain killers (including; opioid agents, NSAIDs or even regional analgesic techniques) have less analgesic effects on them compared to the general population (5-7, 32, 33, 35). Previous studies have demonstrated other clinical problems in opium abusing patients, especially when they are managed for their acute pain status (2, 4, 7, 32, 33, 36). Ketamine, an NMDA antagonist, acts through its specific receptors which are not frequently exposed to drugs abused by these patients before their surgery (17, 34, 37-39). This is possibly one of the main explanations justifying our findings regarding the acceptable effects of ketamine in opium abusers. Results of the present study also demonstrated the opium sparing effects of ketamine used as an adjuvant analgesic to opioids; while its side effects are acceptable. The adjuvant use of ketamine could be an interesting topic for clinicians managing acute pain in these patients.

During recent years, there have been a number of studies stating their concerns regarding the possible unwanted effects of ketamine (40-46). These are usually related to the untoward effects of ketamine on neuro-developmental issues in pediatric patients and not in the adult population. Nonetheless, there are some alerts regarding possible apoptotic effects of ketamine on liver cells, especially in patients exposed to the drug frequently or as an abuse form (41, 47-49). In these studies, the authors are usually 'apprehensive' of the effects of ketamine on infants and children.

There are a number of limitations in our study; first of all, our patients did not include females and some unwanted effects of ketamine are more commonly seen in women (including the emergence reaction). In addition, our patients were only in the orthopedic ward, while other types of procedures that produce postoperative pain (like the GI tract, respiratory tract, or Central Nervous System surgeries) were not involved, and therefore the side effects of ketamine could be more severe in such cases. Overall, our study demonstrated the effectiveness of ketamine's analgesic effects when used as an adjuvant analgesic accompanied with morphine, and as a PCA analgesia in opium abuser patients undergoing orthopedic surgery.
